# Supplementary material for: Electrocorticography reveals thalamic control of cortical dynamics following traumatic brain injury
Source: Commun Biol. 2021 Oct 21;4:1210. doi: 10.1038/s42003-021-02738-2 (PMC8531397; doi:10.1038/s42003-021-02738-2)
Supplement: Supplementary file 2 — Supplementary Information [file 42003_2021_2738_MOESM2_ESM.pdf]

Supplementary Material

Subject 1	Day	TBI (5 days prior)	Day 0 (Implant)	2*	3*	4*	-	-	-	-	-	-	-
	GCS	6	6	3	9	9							
	CRS-R			2	7	10							
Subject 2	Day	TBI	Day 0 (Implant)	1	3	5	7	8*	10*	11*	12*	13*	14*
	GCS		5	3	4	6	6	3	6	9	6	9	8
	CRS-R			0	0	0	0	0	0	2	2	6	7
Subject 3	Day	TBI (10 days prior)	Day 0 (Implant)	1*	2	3*	4*	5*	6*	-	-	-	-
	GCS	3	3	3	3	3	6	5	4				
	CRS-R			0	0	0	0	2	2				
Subject 4	Day	TBI (2 days prior)	Day 0 (Implant)	1*	2*	-	-	-	-	-	-	-	-
	GCS	3	3	6	7								
	CRS-R			10	10								
Subject 5	Day	TBI (1 days prior)	Day 0 (Implant)	1*	2*	3*	4*	-	-	-	-	-	-
	GCS	4	3	6	3	6	6						
	CRS-R			0	2	2	2						

Supplementary Fig. 1. Patient Timelines. CRS-R and GCS of patients relative to injury and implant. Day 0 (shown in orange) corresponds to the day that the depth electrode was implanted. Asterisks mark those days that single pulses of stimulation were administered.

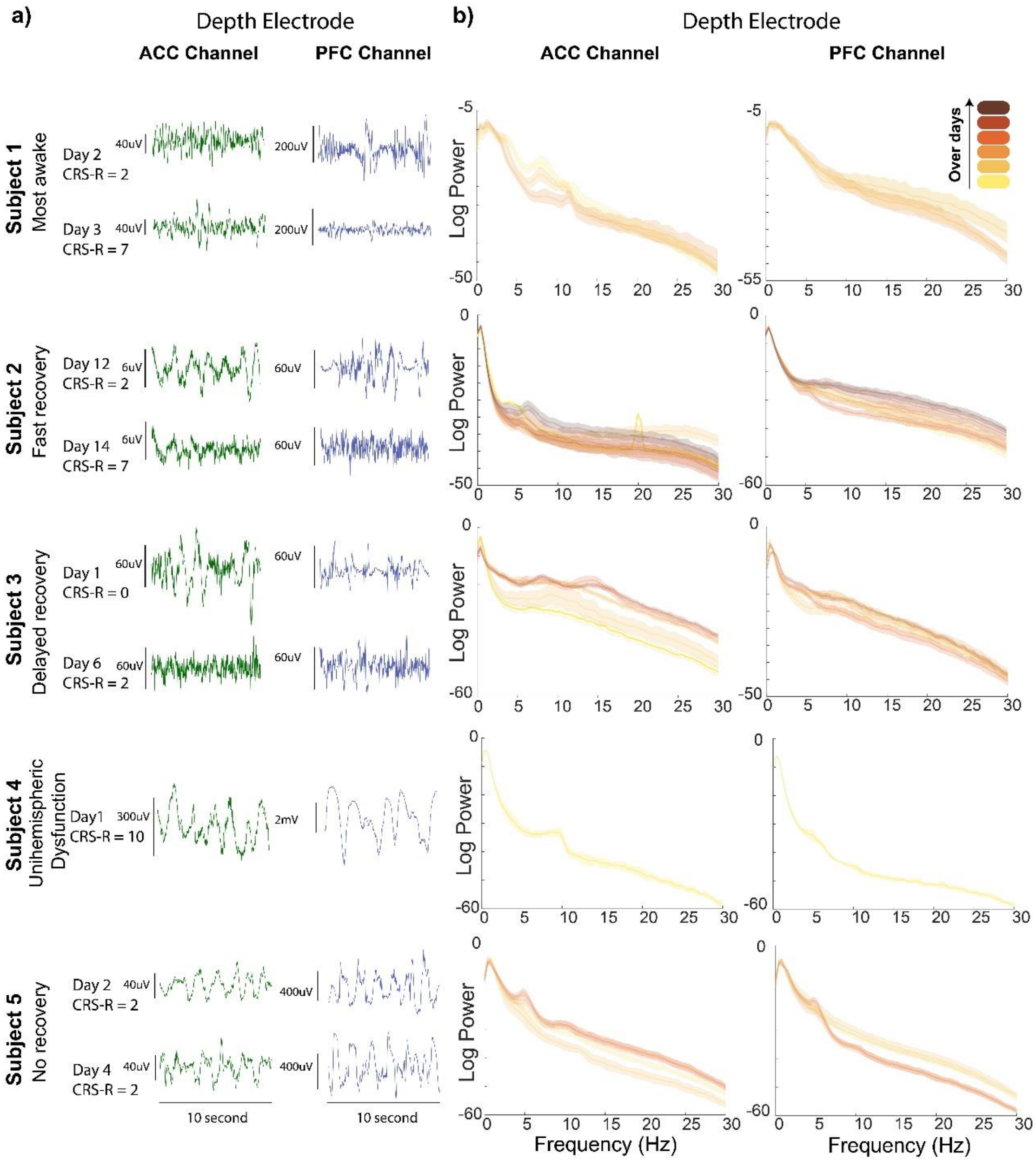

Supplementary Fig. 2. Depth cortical recordings reflect the consciousness level. a) LFP timeseries from depth electrode contacts in PFC and ACC are shown for various days after the injury. **b)** The power spectra of the broadband LFP activity recorded from the depth electrode at PFC and ACC contacts, half an hour after cessation of sedation and before stimulation were calculated for various days after the injury. Here lighter colors are associated with earlier days and darker colors to later days. Power spectra were calculated by dividing the length of the signal (15

min) into one-minute intervals and obtaining the signal power spectra for each interval. These were then averaged to create a mean (thick line) as well as standard deviation (shaded area) for each day.

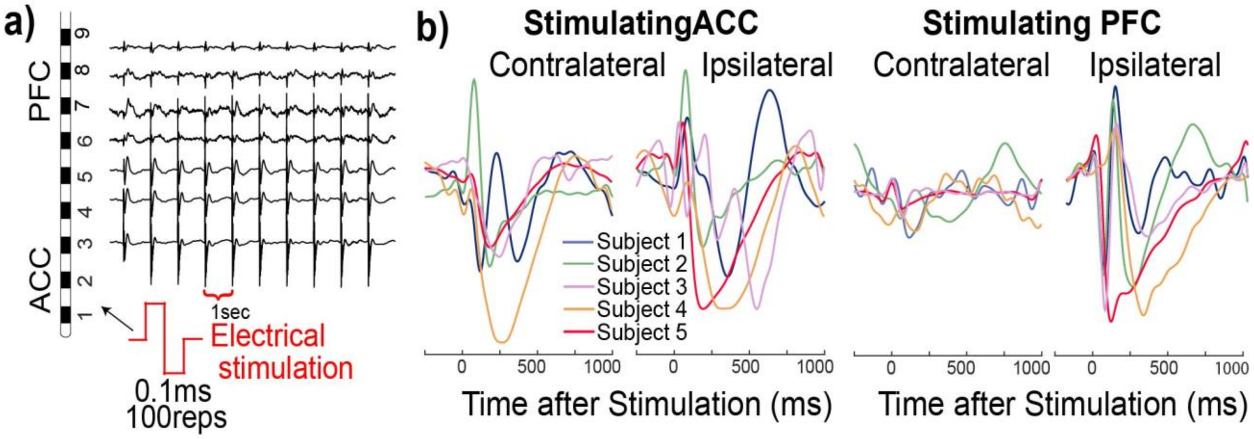

Supplementary Fig. 3. Depth cortical stimulation reveals a hub-like connectivity structure for ACC and local connectivity topology for PFC. a) A typical example of responses to a train of single pulses of electrical stimulation. **b)** Averaged CCEPs for each subject recorded on scalp contacts for ACC (left panel) and PFC (right panel).

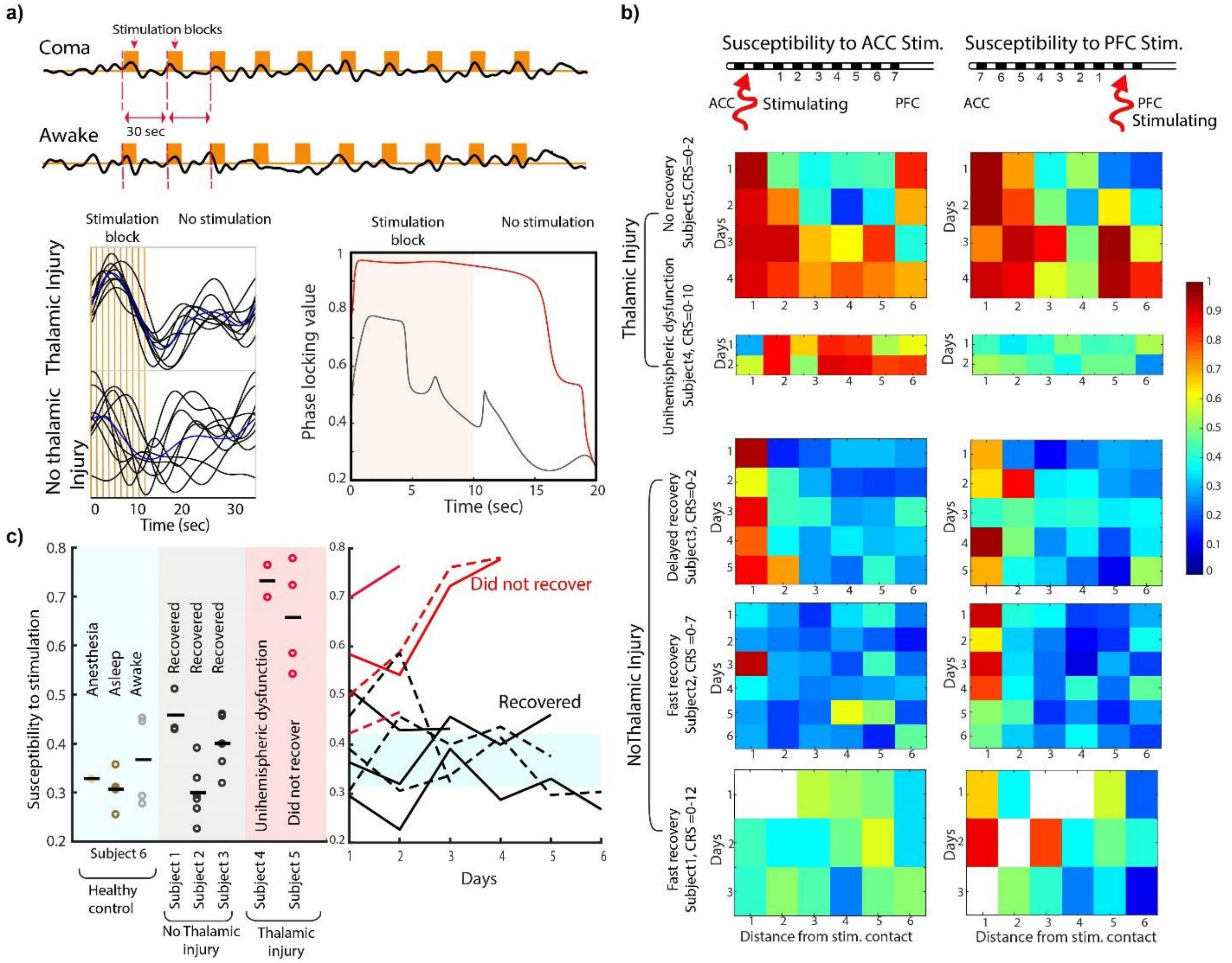

Supplementary Fig. 4. Resilience of the ongoing LFP to trains of single-pulse stimulation is an indication of the functional integrity of cortical networks after traumatic brain injury. a) Top: In comatose patients with thalamic injury, the phase of the low-frequency LFP (low-pass filtered <0.1 Hz, black trace) is highly entrained by trains of repetitive stimuli (orange blocks). Trials are overlaid in the bottom left. The LFP phase for the patient with thalamic injury remained entrained up to 10 seconds after stimulation (bottom right, red line). This entrainment effect either does not exist or rapidly wanes in patients with no thalamic injury (bottom left; blue line on bottom right). **b)** The degree of entrainment to ACC (left) and PFC (right) stimulation reflects the susceptibility of the brain to external stimulation and was quantified by the temporal synchrony of LFP responses across trials. **c)** Left: Susceptibility in patients with thalamic injury is dramatically

higher than in patients with no thalamic injury (Wilcoxon rank-sum test, $p=0.0001$). The healthy control showed a minimal level of susceptibility to stimulation while awake, asleep, and under anesthesia. Right: Susceptibility trend over days (dashed = PFC stimulation, solid = ACC stimulation; blue stripe = the awake-healthy control +/- 1 SD).

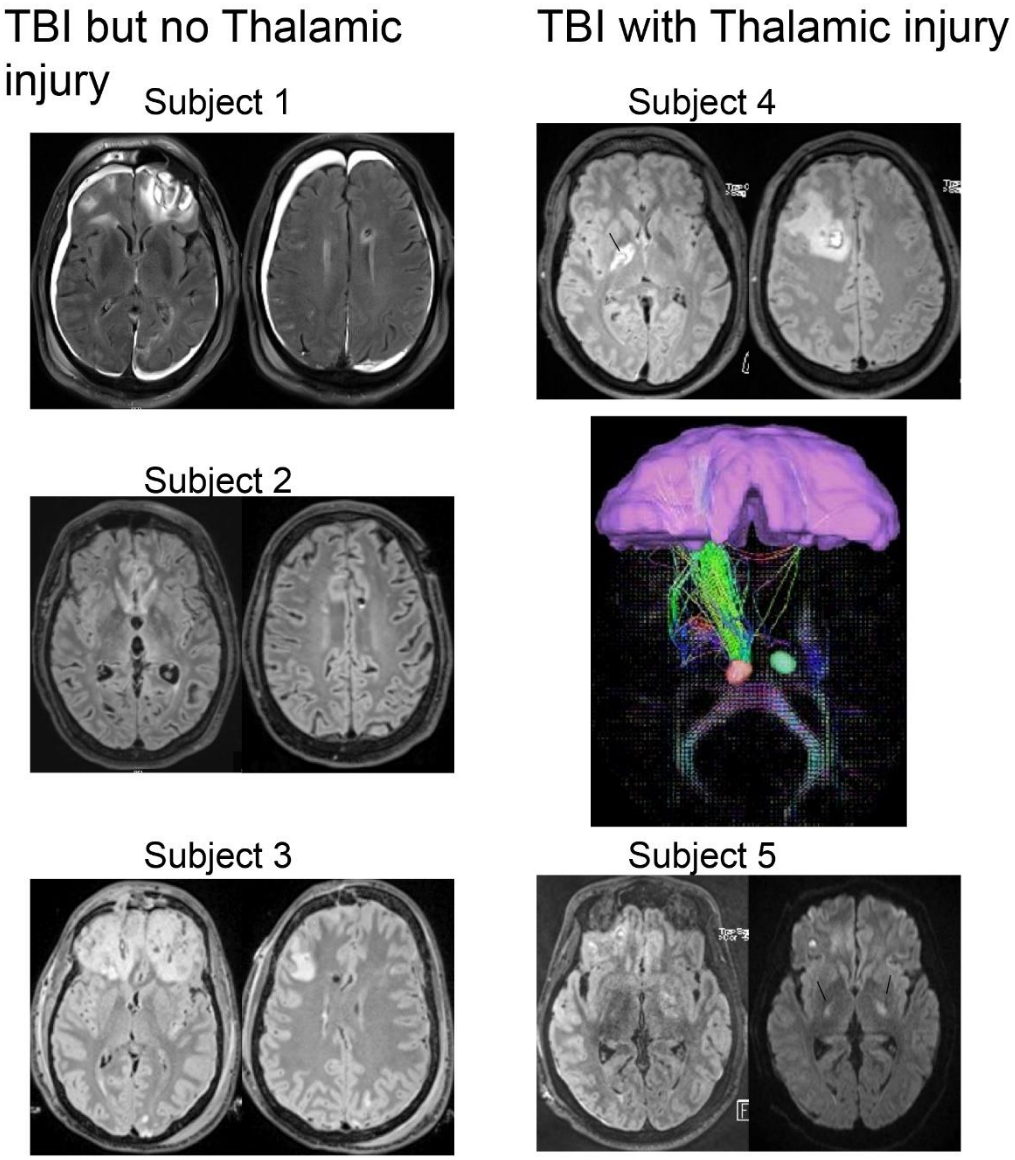

Supplementary Fig. 5. T2-weighted scans from all patients are shown. For Subject 4, DTI from the mediodorsal thalamic nucleus reveals intact left-sided but absent right-sided projections. For Subject 5, the right panel is diffusion-weighted imaging showing injury to projections just anterior to and contiguous with the thalamus (black arrows).

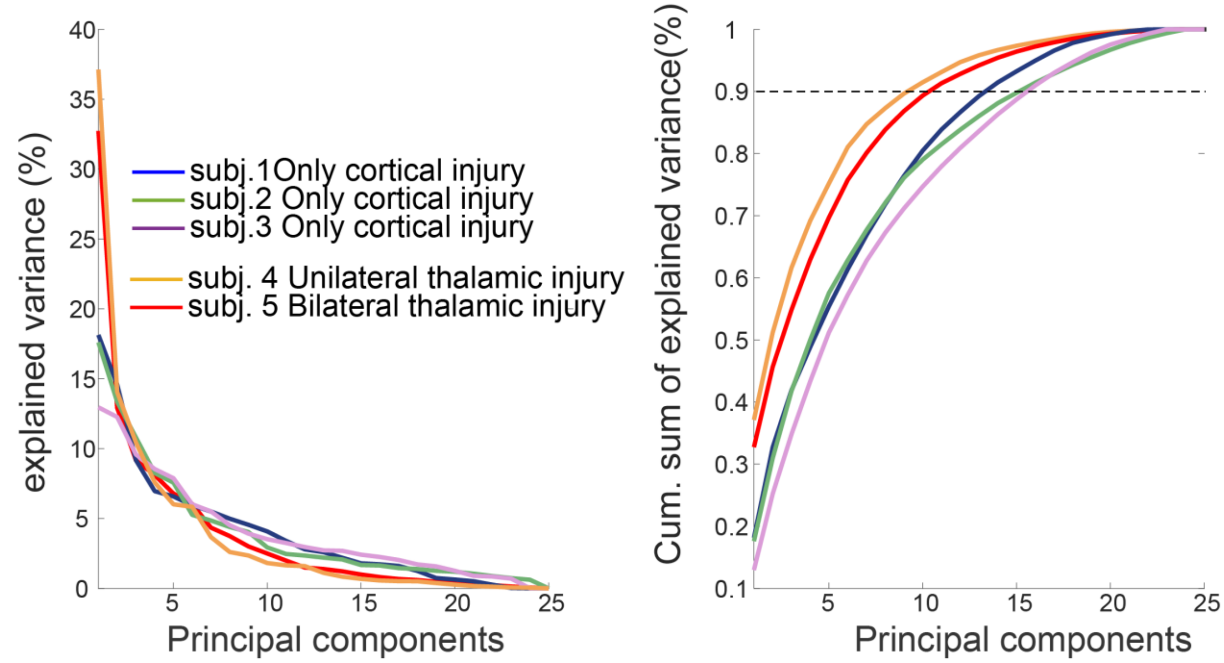

Supplementary Fig. 6. Principal component analysis of cortical activity (all the depth and EEG channels) reveals that patients with extended injuries to the thalamus exhibit low-dimensional cortical activity. We used PCA on a stimulation-free ECoG recording from all scalp and depth channels to identify the minimum number of dimensions needed to explain the data. All patients with intact thalamic projections required more dimensions to explain more of the variance (left panel). A larger number of eigenvalues were required, depending on thalamic injury (right panel, red and yellow lines) or normal thalamic connectivity (blue, green, and purple lines).

Supplementary Note 1

Subject 1

Subject 1 was a 78-year-old man that suffered trauma to the head following a fall down an embankment of about 10 feet. The trauma sustained resulted in a nondisplaced occipital bone fracture extending to the posterior foramen magnum, extensive intracranial hemorrhage without midline shift, cerebral hemorrhagic contusions in the left anterior and inferior frontal lobe, T10 and T11 fractures with trace paraspinal soft tissue hematoma, and a right T10 transverse fracture. Initially, his GCS was 14 (E4V4M6) and his pupils were 2 mm and bilaterally reactive, but he experienced a sharp decline in GCS. After implantation, he required reoperation for a posterior fossa stroke, and rapidly regained consciousness thereafter. His hospital length of stay was 29 days, and he was discharged to TBI rehabilitation with a final GCS of 11.

Subject 2

Subject 2 was a 54-year-old man that suffered traumatic cardiac arrest following a fall from a moped traveling at high speed. The trauma sustained resulted in a massive subarachnoid hematoma, mild ventricular hemorrhage and ventriculomegaly, a left scapula fracture, fracture of the right ribs 1-7, fracture of the left ribs 1-6, right pneumothorax, and pneumomediastinum with a small mediastinal hematoma. Initially, his GCS was 5T (E1V1M3), and his pupils were 2 mm and bilaterally non-reactive. We placed a left-sided electrode rather than a right-sided electrode to maximize scalp availability for possible future procedures, such as a ventriculoperitoneal shunt. Although he followed commands 38 days after injury, his increase in CRS-R was relatively steep (see Supplementary Figure 7). His hospital length of stay was 61 days, and he was discharged to TBI rehabilitation with a final GCS of 11.

Subject 3

Subject 3 was a 49-year-old man who was a pedestrian who was struck by a car in a hit-and-run incident. His injuries included skull base fractures, subarachnoid hematoma, subdural hematoma, intracranial hemorrhage, orbital fractures, and a left humeral fracture. Initially, his GCS was 3 and his pupils were 3-4mm and bilaterally reactive. While he followed commands 51 days after injury, he had a subsequent hemorrhage related to a delayed neurosurgical procedure and ultimately expired 410 days after injury.

Subject 4

Subject 4 was a 25-year-old man who was riding a motorcycle when he collided with a garbage truck. His injuries included TBI, multiple skull fractures, bilateral subarachnoid hemorrhages, subdural hematoma, spinous process fractures, and splenic laceration. Initially his GCS was 3, and his right and left pupils were 3 & 4mm respectively, both non-reactive to light. He recovered consciousness and was discharged to rehab on hospital day 39.

Subject 5

Subject 5 was a 38-year-old man that was riding a bicycle without a helmet when he was struck by a car. The trauma sustained resulted in a right open depressed skull fracture, subarachnoid hematoma, and diffuse axonal injury. Initially, his GCS was 4 (E1V1M2) and his pupils were anisocoric. He never recovered consciousness. His hospital length of stay was 196 days, and he ultimately expired from his injuries.

Control Subject

The control subject was a 24-year-old woman with refractory epilepsy who underwent an extensive stereo-EEG depth electrode implant with bitemporal and bifrontal coverage, including bilateral electrodes which spanned prefrontal cortex and dorsal anterior cingulate, in an identical anatomical location to the study patients. No seizures were identified during the period of recording, however.

Supplementary Table 1. Inclusion and Exclusion Criteria.

Inclusion Criteria:	1. Age $\geq$18
	2. Traumatic brain injury with GCS $\leq$ 8
Exclusion Criteria:	1. Major brain structural abnormalities (excepting hemicraniectomy) that preclude between-subjects comparison
	2. No legally authorized representative (LAR) available to provide proxy informed consent
	3. Known pre-injury neurological disease
	4. Any other medical condition that, in the judgment of the investigator, makes participation in the study unsafe.
	5. Females of childbearing potential who are pregnant

Supplementary Table 2. Patients 1-5 Sedative Medication Regimens

Sedative Medications	Subject 1	Subject 2	Subject 3	Subject 4	Subject 5	Control
	Dexmedetomidine Cont. IV	Dexmedetomidine Cont. IV	x	x	Dexmedetomidine Cont. IV	x
	Fentanyl Cont. IV	Fentanyl Cont. IV	Fentanyl Cont. IV	Fentanyl Cont. IV	Fentanyl Cont. IV	x
	Fentanyl PRN IV Push	x	Fentanyl PRN IV Push	x	Fentanyl PRN IV Push	x
	x	Midazolam Cont. IV	Midazolam Cont. IV	x	x	x
	x	x	Midazolam PRN IV Push	x	Midazolam PRN IV Push	x
