# Supplementary material for: Electrocorticography reveals thalamic control of cortical dynamics following traumatic brain injury
Source: Commun Biol. 2021 Oct 21;4:1210. doi: 10.1038/s42003-021-02738-2 (PMC8531397; doi:10.1038/s42003-021-02738-2)
Supplement: Supplementary file 3 — Description of Additional Supplementary Files [file 42003_2021_2738_MOESM3_ESM.pdf]

Description of Additional Supplementary Files

File name: Supplementary Data 1

Description: Source data for main text figures. Data used to generate charts from Figures 1-4 are included. Each figure’s data is in the corresponding sheet of the Excel file.
