# Supplementary material for: Electrocorticography reveals thalamic control of cortical dynamics following traumatic brain injury
Source: Commun Biol. 2021 Oct 21;4:1210. doi: 10.1038/s42003-021-02738-2 (PMC8531397; doi:10.1038/s42003-021-02738-2)
Supplement: Supplementary file 5 — Reporting Summary [file 42003_2021_2738_MOESM5_ESM.pdf]

Corresponding author(s): Charles Mikell
Last updated by author(s): Aug 23, 2021

Reporting Summary

Nature Portfolio wishes to improve the reproducibility of the work that we publish. This form provides structure for consistency and transparency in reporting. For further information on Nature Portfolio policies, see our Editorial Policies and the Editorial Policy Checklist.

Statistics

For all statistical analyses, confirm that the following items are present in the figure legend, table legend, main text, or Methods section.

n/a	Confirmed	
☐	☒	The exact sample size (*n*) for each experimental group/condition, given as a discrete number and unit of measurement
☐	☒	A statement on whether measurements were taken from distinct samples or whether the same sample was measured repeatedly
☐	☒	The statistical test(s) used AND whether they are one- or two-sided<br>*Only common tests should be described solely by name; describe more complex techniques in the Methods section.*
☐	☒	A description of all covariates tested
☐	☒	A description of any assumptions or corrections, such as tests of normality and adjustment for multiple comparisons
☐	☒	A full description of the statistical parameters including central tendency (e.g. means) or other basic estimates (e.g. regression coefficient) AND variation (e.g. standard deviation) or associated estimates of uncertainty (e.g. confidence intervals)
☒	☐	For null hypothesis testing, the test statistic (e.g. *F*, *t*, *r*) with confidence intervals, effect sizes, degrees of freedom and *P* value noted<br>*Give P values as exact values whenever suitable.*
☒	☐	For Bayesian analysis, information on the choice of priors and Markov chain Monte Carlo settings
☒	☐	For hierarchical and complex designs, identification of the appropriate level for tests and full reporting of outcomes
☒	☐	Estimates of effect sizes (e.g. Cohen's *d*, Pearson's *r*), indicating how they were calculated

Our web collection on statistics for biologists contains articles on many of the points above.

Software and code

Policy information about availability of computer code

Data collection

We used NATUS software to obtain the electrophysiological recordings in .edf format.

Data analysis

• All code was written in MATLAB.

• We utilized a combination of FieldTrip toolbox software and custom code to convert the .edf files to .mat format and cut, organize and structure the recorded local field potential (LFP).

• We wrote custom code in MATLAB to demean and re-reference the data (bipolar re-referencing).

• filtfilt MATALB function was used to filter these signals and MATLAB's norm function to normalize. Power spectral density calculations analysis were done using pwelch function in MATALB (default settings, 50% overlap).

• To compute the LZ complexity we wrote custom code; but also compared to the open source code available online (LZ Complexity method https://www.mathworks.com/matlabcentral/fileexchange/38211-calc_lz_complexity
cite:
Quang Thai(2021). calc_lz_complexity (https://www.mathworks.com/matlabcentral/fileexchange/38211-calc_lz_complexity), MATLAB Central File Exchange. Retrieved February 2, 2021.)

• To compute zero crossing we wrote custom code.

• We utilized PhaseSpaceReconstruction function in MATLAB to reconstruct the phase space underlying the observed dynamics. This function estimates the embedding dimension, while we used autocorrelation (using xcorr function in MATLAB) to estimate the appropriate time lag.

• We also performed principal component analysis using MATLAB's built-in PCA function.

For manuscripts utilizing custom algorithms or software that are central to the research but not yet described in published literature, software must be made available to editors and reviewers. We strongly encourage code deposition in a community repository (e.g. GitHub). See the Nature Portfolio guidelines for submitting code & software for further information.

Data

Policy information about availability of data

All manuscripts must include a data availability statement. This statement should provide the following information, where applicable:

- Accession codes, unique identifiers, or web links for publicly available datasets
- A description of any restrictions on data availability
- For clinical datasets or third party data, please ensure that the statement adheres to our policy

All source data for our figures are included as Supplementary Data files. Original recordings and images recordings, images are available upon reasonable request.

Field-specific reporting

Please select the one below that is the best fit for your research. If you are not sure, read the appropriate sections before making your selection.

☒ Life sciences ☐ Behavioural & social sciences ☐ Ecological, evolutionary & environmental sciences

For a reference copy of the document with all sections, see nature.com/documents/nr-reporting-summary-flat.pdf

Life sciences study design

All studies must disclose on these points even when the disclosure is negative.

Sample size	Based on the availability. In this study we report depth electrophysiological recordings in TBI comatose population, which is extremely rare and precious. To the best of our knowledge this is the first report of such study.
Data exclusions	No data exclusion.
Replication	This is a pilot study showing the effect. We need to recruit more TBI patients in future to test the reproducibility of the presented findings. However, for our limited number of patients (n=5) we had many trials (n=100) over several days (2-6 days) and results were consistent.
Randomization	Not relevant.
Blinding	They were not blinded.

Reporting for specific materials, systems and methods

We require information from authors about some types of materials, experimental systems and methods used in many studies. Here, indicate whether each material, system or method listed is relevant to your study. If you are not sure if a list item applies to your research, read the appropriate section before selecting a response.

Materials & experimental systems

n/a	Involved in the study	
☒	☐	Antibodies
☒	☐	Eukaryotic cell lines
☒	☐	Palaeontology and archaeology
☒	☐	Animals and other organisms
☐	☒	Human research participants
☒	☐	Clinical data
☒	☐	Dual use research of concern

Methods

n/a	Involved in the study	
☒	☐	ChIP-seq
☒	☐	Flow cytometry
☐	☒	MRI-based neuroimaging

Human research participants

Policy information about studies involving human research participants

Population characteristics	Population characteristics are described in Table 1.
Recruitment	There were no bias in the recruitment process. Our sample represents the demographics of TBI patients in Long Island.

Ethics oversight	The study was approved by Stony Brook Committee on Research Involving Human Subjects.

Note that full information on the approval of the study protocol must also be provided in the manuscript.

Magnetic resonance imaging

Experimental design

Design type	Structural MRI
Design specifications	To identify structural pathology in the brain.
Behavioral performance measures	No relevant measures.

Acquisition

Imaging type(s)	Structural
Field strength	3T; Subjects 3 and 5 were acquired at 1.5 T
Sequence & imaging parameters	These images were obtained as clinical (not research) MRIs and represent the best images that could be obtained under the circumstances. Volumetric Siemens FLAIR (fluid-attenuated inversion recovery) sequences were used for all patients. Matrix size was 256x256. Slice thickness/gap was 1/0 mm. Flip angle = 120, TE/TR = 335/5000 ms (exception: TR=4500 for subject 1), TI=1800 ms, pixel size = 0.9375 mm. Scans were typically acquired in the sagittal plane and resampled to axial. For subject 4, the shown diffusion tensor images were acquired using an EPI sequence with flip angle = 90, TE/TR = 90/5400 ms, slice thickness/gap = 4/0 mm, 32 diffusion direction, single shell scheme with b=1000. For subject 5, a diffusion-weighted EPI image is also shown (TE/TR = 90/10300 ms; flip angle = 90, b= acquisition matrix = 200x200 and slice thickness/gap was 4/0 mm).
Area of acquisition	Whole brain

Diffusion MRI ☒ Used ☐ Not used

Parameters	32 directions, single shell, b = 1000, no cardiac gating.

Preprocessing

Preprocessing software	N/A
Normalization	N/A
Normalization template	N/A
Noise and artifact removal	N/A
Volume censoring	N/A

Statistical modeling & inference

Model type and settings	N/A
Effect(s) tested	N/A

Specify type of analysis: ☐ Whole brain ☐ ROI-based ☐ Both

Statistic type for inference (See Eklund et al. 2016)	N/A
Correction	N/A

Models & analysis

n/a	Involved in the study
☒	☐ Functional and/or effective connectivity
☒	☐ Graph analysis
☒	☐ Multivariate modeling or predictive analysis
